# Open versus arthroscopic repair of the triangular fibrocartilage complex: a systematic review

**DOI:** 10.1186/s40634-018-0120-1

**Published:** 2018-03-13

**Authors:** Jonny K. Andersson, Martina Åhlén, Daniel Andernord

**Affiliations:** 1 Department of Hand Surgery, Sahlgrenska University Hospital, SE-413 45 Göteborg, Sweden; 2 Department of Orthopaedics, Institute of Clinical Sciences, The Sahlgrenska Academy, University of Gothenburg, Göteborg, Sweden; 3 Vårdcentralen Gripen, Karlstad, Sweden; 4 Centre for Clinical Research, County Council of Värmland, Karlstad, Sweden

**Keywords:** Triangular fibrocartilage complex, Open repair, Arthroscopic repair, Systematic review

## Abstract

**Background and purpose:**

To investigate the outcome of open versus arthroscopic repair of injuries of the triangular fibrocartilage complex (TFCC).

**Methods:**

An electronic literature search of articles published between January 1, 1985, and May 26, 2016, in PubMed, Embase, and the Cochrane Library was carried out in May 2016 and updated in March and December 2017. Studies comparing open and arthroscopic repair of TFCC injury with a mean follow up of more than 1 year were eligible for inclusion. The Preferred Reporting Items for Systematic Reviews and Meta-Analyses (PRISMA) checklist guided the extraction and reporting of data. The methodological quality of the included articles was assessed with the Cochrane Collaboration’s tool for assessing risk of bias. The primary outcome measure was the rate of postoperative distal radioulnar joint (DRUJ) re-instability. Secondary outcome measures were range of motion (ROM), grip strength, residual pain, functional wrist scores and the rates of complications and re-operations.

**Results:**

A total of 868 articles were identified by the electronic search. After duplicate removal and subsequent study selection, a total of two studies were included in this systematic review. The methodological quality of the included articles displayed risks of bias. There was no difference in DRUJ re-instability between open and arthroscopic repair of the TFCC. There were no differences in obtained postoperative ROM, grip strength or values in functional outcome scores, between open and arthroscopic TFCC repair in the two included studies, except for the Disability of the Arm Shoulder and Hand (DASH) questionnaire - in favor of arthroscopic surgery - in one of the included studies.

**Conclusions:**

This systematic review shows comparable results between open and arthroscopic repair of the TFCC, in terms of DRUJ re-instability and functional outcome scores. There is insufficient evidence to recommend one technique over the other in clinical practice. There is an immense lack of comparison studies with high level of evidence in the area of wrist ligament repair and reconstruction, including TFCC-injuries and DRUJ-instability.

## Review

### Introduction

Injury to the triangular fibrocartilage complex (TFCC) is the most common wrist ligament injury. More than 40% of displaced distal radial fractures are associated with a TFCC injury (Geissler et al. 1996; Andersson and Axelsson 2011; Lindau et al. 1997; Richards et al. 1997; Scheer and Adolfsson 2011). Injury to the TFCC is sometimes associated with instability of the distal radioulnar joint (DRUJ). Lindau et al. (2000) demonstrated that instability of the DRUJ is a negative factor in terms of clinical outcome after distal radial fractures in young patients, independent of radiographic findings. Isolated TFCC injury also occurs as a result of rotational trauma to the wrist. In patients with post-traumatic wrist pain but normal standard radiographs, 42% were found to have TFCC injuries (Adolfsson 1994).

**Fig. 1:**
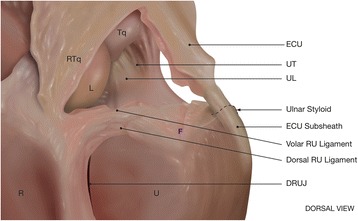
TFCC anatomy. Palmar and dorsal radio-ulnar (RU) ligaments, ulnocarpal ligaments (UL, UT) are displayed. (ECU = extensor carpi ulnaris, DRUJ = distal radio-ulnar joint, R = radius, U = ulna, L = lunate, Tq = triquetrum, RTq = radiotriquetral ligament, F = ulnar fovea)

The anatomical location of traumatic lesions to the TFCC is one of several factors that impact the surgical treatment (Osterman 1990; Palmer 1990). Several anatomical structures stabilize the DRUJ, of which the TFCC and especially its foveal insertion is the most important (Fig. 1) (Haugstvedt et al. 2006). Dorso-ulnar wrist pain is present in all types of TFCC injury. Concomitant DRUJ instability is present mainly in injuries with avulsion from the foveal attachment of the ulna, classified as 1B injuries according to Palmer (1989) or class 2–3, according to Atzei and Luchetti (2011).

**Fig. 2:**
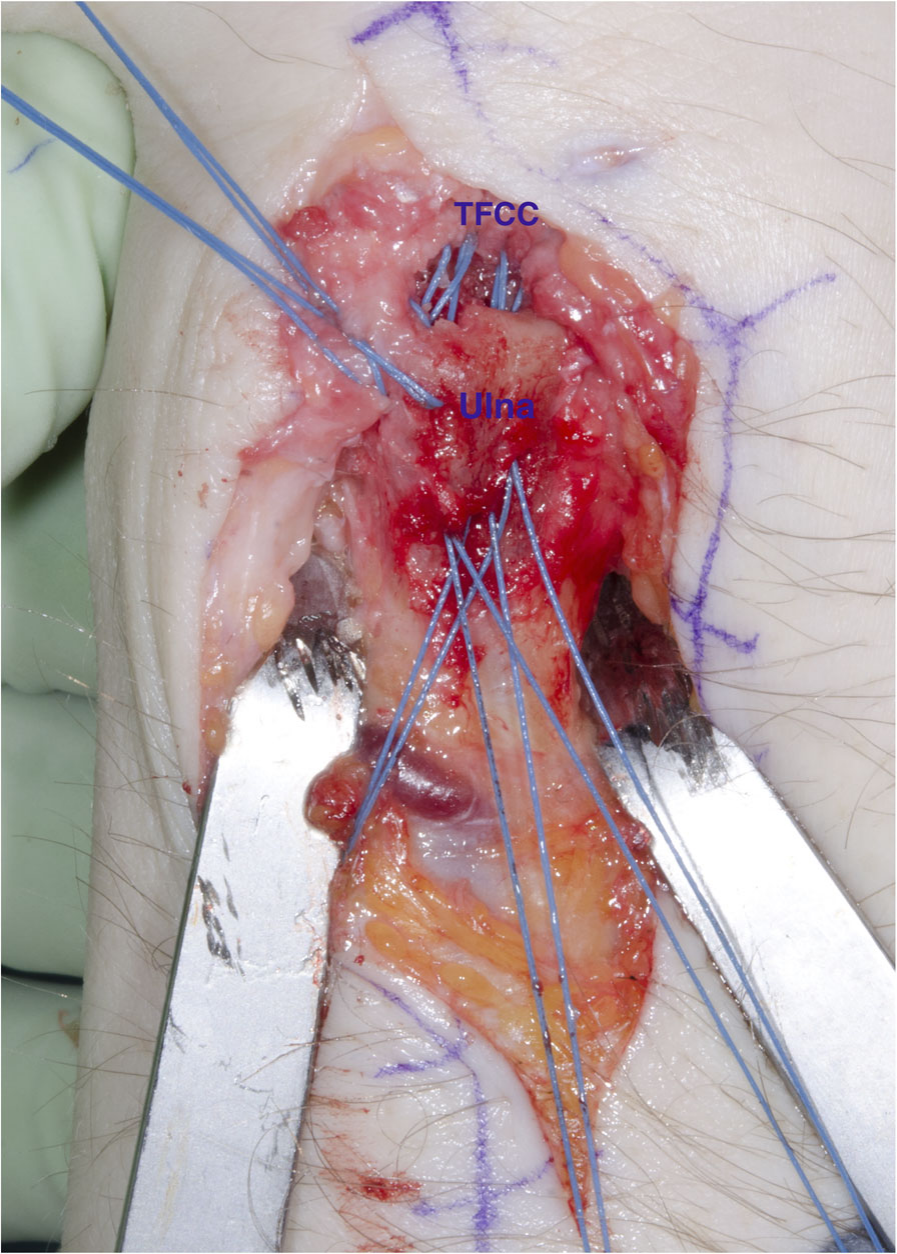
Open re-insertion of the TFCC with five transosseous sutures (TFCC = triangular fibrocartilage complex, Ulna = distal ulna). Left wrist

**Fig. 3:**
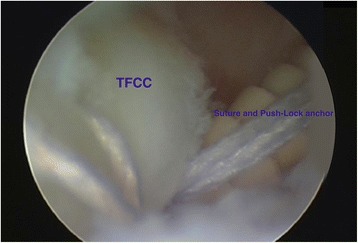
Arthroscopic re-insertion of the TFCC with push-lock suture anchor (Arthrex®). Right wrist

Open repair of TFCC tears was one of the first established treatment options for acute TFCC injuries (Cooney et al. 1994). However, ulnar-sided TFCC lesions, Palmer type 1B, which are predisposed to cause DRUJ instability can nowadays be treated using either an open approach (Fig. 2) (Cooney et al. 1994; Garcia-Elias et al. 2003) or an arthroscopically assisted procedure with transosseous sutures (Atzei et al. 2008; Bednar and Osterman 1994). The TFCC can also be repaired with open surgery using bone anchors or arthroscopically using push-lock anchors (Fig. 3). Radial-sided TFCC lesions, Palmer type 1D, are also predisposed to cause DRUJ instability and are best treated by re-insertion to the radius (Carlsen et al. 2009; Tang et al. 2013). When re-insertion of the TFCC is not possible, anatomic reconstruction (Adams 2000) is the method of choice. Proponents of arthroscopic repair of the TFCC have claimed better visualization of the injuries and final range of motion (ROM) and less complications (Bednar and Osterman 1994). In contrast, some authors suggest that only open surgery can restore the foveal attachment of the TFCC (Sagerman and Short 1996; Trumble et al. 1996). Reports of different surgical techniques are common. There are still, however, no consensus or recommendations on whether TFCC injuries should be treated with open or arthroscopic surgery. The purpose of this systematic review was to investigate and compare the outcome of open versus arthroscopic repair of injuries to the TFCC. The hypothesis was that arthroscopic technique of TFCC repair is comparable with traditional open techniques.

### Methods

#### Protocols

This systematic review was conducted according to the Preferred Reporting Items for Systematic Reviews and Meta-Analyses (PRISMA) statement (Moher et al. 2009). The quality assessment of included studies was performed using the Cochrane Collaboration’s tool for assessing risk of bias (Higgins and Green 2008).

#### Eligibility criteria

Studies with comparison of open vs arthroscopic repair of TFCC injuries with minimum 12 months follow up, published in the English language between January 1, 1985 and May 26, 2016 - finally updated to December 27, 2017 - were eligible for inclusion. Exclusion criteria were pediatric populations, cadaveric or animal studies, study protocols, and re-injury and revision surgery, as well as studies of instrumentation, surgical technique and studies evaluating anatomical reconstructions of the DRUJ (e g Adams) and suture only of TFCC to capsule (not re-insertion). Expert opinions and case reports were also excluded.

#### Information sources and search

A systematic electronic search of PubMed, Embase, and the Cochrane Library was carried out on May 26, 2016 by an expert in electronic search strategies at the Sahlgrenska University Hospital Library. The systematic electronic search was updated on March 21, 2017, and on December 27, 2017, in order to identify newly published studies that were eligible for inclusion. Corresponding authors were not contacted for additional information. The complete electronic search strategies are described in the Appendix.

#### Study selection

The first (JKA) and second author (MÅ) performed the study selection, which was validated in duplicate. All articles generated by the electronic search were screened by reading the title and abstract. If initial screening failed to provide sufficient information for the purpose of inclusion or exclusion, the full text article was always assessed.

#### Data collection process and data items

Data extraction was performed according to the PRISMA checklist (Moher et al. 2009) with the use of a standardized extraction sheet. Data items obtained from the included articles were as follows: participants, interventions, comparisons, outcomes, study design and setting (PICOS), allocation, sample size, and possible bias.

#### Assessment of quality and risk of bias

The methodological quality and risk of bias of the included articles was assessed by use of the Cochrane Collaboration’s tool for assessing risk of bias (Higgins and Green 2008), independently by the second author (MÅ) and the senior author (DA). The quality assessment included: random sequence generation, allocation concealment, blinding of participants, personnel and outcome assessment, incomplete data outcome and selective reporting.

#### Outcome measures

The primary outcome measure was the risk of postoperative distal radioulnar joint (DRUJ) re-instability. DRUJ laxity is best tested with the forearm held in neutral rotation by the examiner, who stabilises the hand and the distal radius with a firm grip to make them one unit (Mrkonjic et al. 2012). Then, using the other hand, the examiner forces the ulna as the second unit in a dorsal/palmar direction, relative to the stabilised unit of the hand and radius. The laxity of the DRUJ is always compared with that of the uninjured wrist. A risk of post-operative DRUJ re-instability of less than 20% was considered as clinically acceptable in patients with TFCC injury with concomitant DRUJ instability.

Secondary outcome measures were range of motion (ROM), grip strength, complications, re-operation and functional wrist scores (Modified Mayo Wrist Score [MMWS], Disability of the Shoulder, Arm and Hand [DASH] and Patient-rated Wrist Evaluation [PRWE]). The Modified Mayo Wrist Score (MMWS) (Cooney et al. 1987) consists of a total of 100 points, which are divided among the physician’s assessment of pain (25 p), active flexion/extension arc (25 p) and grip strength (25 p) as a percentage of the opposite side, and the ability to return to regular employment or activities (25 p). For example, pain is rated as none (25 p), mild (20 p), moderate (10 p) or severe (0 p) by the examiner, based on the patient’s subjective description. An excellent score is defined as 90–100 p, good is 80–90, fair is 65–79 and poor 0–64. The DASH (Disability of Shoulder, Arm, and Hand questionnaire) (Hudak et al. 1996) aims to capture the patient’s own perception of their upper extremity function as a single functional unit. The questionnaire contains 30 items; 21 evaluating difficulty with specific tasks, five evaluating symptoms (pain – two modalities; numbness; stiffness; weakness) and one each evaluating social function, work function, sleep and confidence. The score is scaled 0–100, with higher scores indicating *worse* upper-extremity function. Jester et al. (2005) reported a mean DASH score of 13 points +/− 11 in an asymptomatic normal population. The PRWE (Patient-rated wrist evaluation) questionnaire (JC et al. 1998) is a self-administrated questionnaire that is intended to provide a tool for quantifying patient-rated wrist pain and disability in terms of assessing the outcome after distal radius fractures. The score consists of two equal weighted domains – pain and function. The total score ranges from 0 (normal wrist) to 150 (worst possible). The DASH has good validity, reliability and responsiveness (Changulani et al. 2008). The PRWE also has good reliability and responsiveness, but fair validity (Changulani et al. 2008). A rate of at least 80% good-excellent results was considered as acceptable in terms of functional outcomes.

### Results

#### Study characteristics

**Fig. 4:**
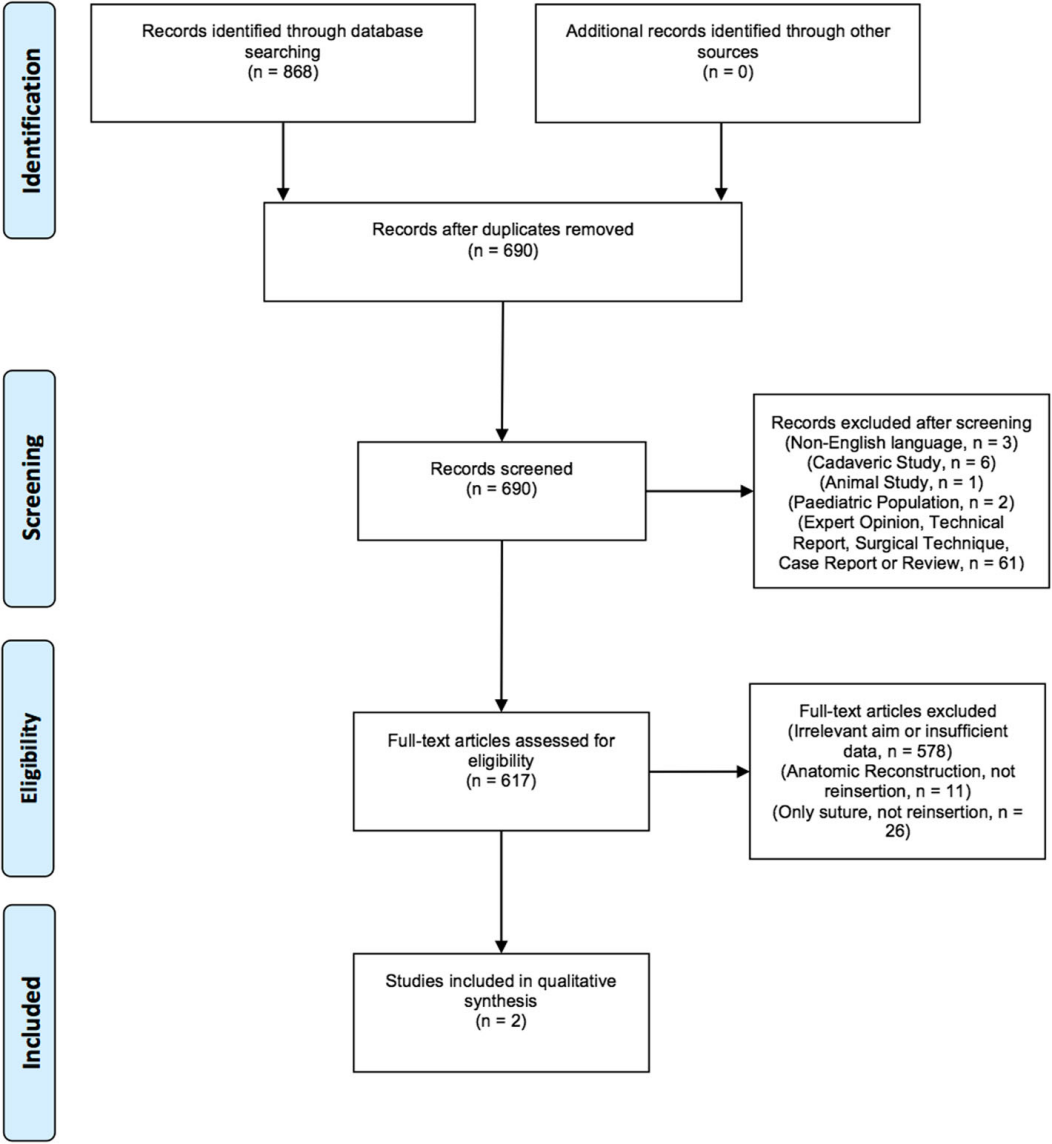
Flow diagram of inclusion and exclusion after the systematic electronic search performed on May 26, 2016. Complete search strategies including the updated searches on March 21, 2017 and December 27, 2017 are shown in the Appendix. The updated electronic searches performed on March, 2017 and December, 2017 did not generate any new studies eligible for inclusion

A total of 868 articles were identified by the electronic search, of which 178 articles were duplicates. After removal of duplicates and subsequent study selection, a total of eight topic-specific articles (Gong et al. 2015; Atzei et al. 2015; Iwasaki et al. 2011; Kim et al. 2013; Seo et al. 2016; Shinohara et al. 2013; Anderson et al. 2008; Luchetti et al. 2014) were identified. Six were case series of open (Gong et al. 2015) or arthroscopic repair (Atzei et al. 2015; Iwasaki et al. 2011; Kim et al. 2013; Seo et al. 2016; Shinohara et al. 2013) of the TFCC. A total of two articles (Anderson et al. 2008; Luchetti et al. 2014) with relevant study design and outcome measures comparing open vs arthroscopic repair of the TFCC were identified and subsequently included in this systematic review. Figure 4 shows the flowchart of inclusion and exclusion of studies. In total, 63 patients underwent open surgery and 62 patients underwent arthroscopic procedures.

Two other studies (Chou and Lee 2001; Nakamura et al. 2011) reporting on both open and arthroscopic techniques were identified by the electronic search. Chou and Lee (2001) reported preoperative and 1-year postoperative MMWS from 17 TFCC sutures of which only three were performed by an arthroscopic technique and no comparisons were made between the two techniques. Nakamura (2011), classified as level of evidence V, reported on surgical techniques and had insufficient data and no preoperative data. Both of these studies were excluded in this systematic review, according to the inclusion and exclusion criteria.

The updated electronic searches performed on March 21, 2017 and on December 27, 2017, did not generate any new studies eligible for inclusion (Table 1).

**Table 1:** Study characteristics (DRF = distal radius fracture, o = open TFCC repair, a = arthroscopic TFCC repair)

Author	Design	Level of Evidence	Sample size	Follow up (months in mean)	Mean age / Male (%)	Concomitant injuries or surgical procedures
Anderson et al. 2008	Prospective Cohort Study	III	39 (o) 37 (a)	43	35(o)-32 (a) / 44 (o)-49 (a)	16% older DRF
Luchetti et al., 2014	Prospective Cohort Study	III	24 (o) 25 (a)	31	32(o)-33(a) / 38 (o)-52(a)	47% older DRF 21% Wafer- resection in open

The mean age was comparable between patients who underwent open and arthroscopic surgery. Arthroscopic surgery was performed slightly more often in men, compared with open surgery. The sample size (mean *n* = 32 in open surgery and *n* = 31 in arthroscopic) was small and the follow up was short term (mean 37 months) in the two included studies.

#### Outcome measures

The outcome measures are displayed in Table 2.

**Table 2:** Outcome measures (Open = open repair of TFCC, a-scop = arthroscopic repair of the TFCC, DRUJ = distal radio-ulnar joint, ECU = extensor carpi ulnaris, VAS = Visual Analog Scale, MMWS = Modified Mayo Wrist Score, DASH = Disability of the Shoulder, Arm and Hand, PRWE = Patient-rated Wrist Evaluation)

Study	DRUJ-re-instabil. (%)	ECU-tendinitis (%)	Neuroma (%)	Pain postop	Good or excellent MMWS (%)	Funct. score (MMWS, DASH, PRWE)
Anderson et al. 2008	21 (open) 14 (a-scop)	26 (open) 11 (a-scop)	36 (open) 22 (a-scop)	- *VAS Postop:* 1.5 (±0.4) (open) 2.6 (±0.9) (a-scop)	*MMWS:* 66 (± 14) *preop* = > 71 (± 25) *postop* (open). 64 (± 15) *preop* = > 71 (± 25) *postop* (a-scop).	*DASH: Postop:* 17 (± 4) (open) 21 (± 6) (a-scop) *PRWE: Postop:* 29 (± 7) (open) 41 (± 11) (a-scop)
Luchetti et al. 2014	17 (open) 4 (a-scop)	–	0 (open) 0 (a-scop)	- *VAS Postop:* 1 (SD 2) *-at rest* 4 (SD 2) *-at stress* (open). 1 (SD 1) *-at rest* 3 (SD 3) -at *stress* (a-scop).	*MMWS*: 48 (SD 16) *preop* = > 78 (SD 17) *postop* (open). 47 (SD 13) *preop* = > 81 (SD 22) *postop* (a-scop).	*DASH*: 58 (SD 23) *preop* = > 36 (SD 20) *postop* (open). 39 (SD 21) *preop* = > 18 (SD 16) *postop* (a-scop). *PRWE*: 69 (SD 29) *preop* = > 42 (SD 29) *postop* (open). 54 (SD 20) *preop* = > 23 (SD 18) *postop* (a-scop).

One paper (Anderson et al. 2008) did not reach the predetermined cutoff value, < 20%, for the primary outcome measure (DRUJ re-instability) in terms of open surgery, but they did reach it in terms of arthroscopic surgery. Both included papers (Anderson et al. 2008; Luchetti et al. 2014) did not reach the predetermined cutoff value for the secondary outcome measure (> 80% excellent-good results, MMWS) both in terms of open surgery. Anderson et al., did not reach the predetermined cutoff value for functional outcome in arthroscopic surgery either. Anderson et al. (2008) showed no statistical difference in functional scores (MMWS, DASH, PRWE) after open versus arthroscopic TFCC repair.

#### Treatment-related adverse events, reoperations and complications

Although not statistically significant, Anderson et al., described an increased rate of postoperative superficial ulnar nerve pain in the open group (36%) compared with the arthroscopic group (22%). Extensor carpi ulnaris (ECU) tendonitis was reported in an increased – although not statistically significant – rate in open surgery (26%) compared with arthroscopic surgery (11%), in the study by Anderson et al. (2008). No nerve lesions or ECU tendonitis were reported in either group in the study by Luchetti et al. (2014). None of the two included studies reported any information about the rate of patients having residual pain, but both studies showed similiar VAS values in terms of mean values post-operatively. There were no statistically significant differences in obtained postoperative range of motion, grip strength or pain (VAS) between the open and arthroscopic group in the two included studies.

After TFCC repair, 13 out of 75 patients required reoperation for DRUJ instability – eight (21%) in the open group and five (14%) in the arthroscopic group, in the study by Anderson et al. (2008). Anderson et al. (2008) displayed a 4.95 times higher re-operation rate among female patients compared with men (*p* = 0.003). Luchetti et al. (2014) found that DRUJ instability recurred in five patients in total – four in open technique (17%) and one in arthroscopic (4%). Luchetti et al. (2014) also noted no significant postoperative difference between open and arthroscopic reinsertion in outcome measures, except for DASH (*p* < 0.001) and PRWE (*p* < 0.01), which was significantly better among patients operated on by arthroscopic surgery. Anderson et al. (2008) found no significant statistical difference between open and arthroscopic groups based on the DASH or PRWE.

In total, twenty patients in the study by Anderson et al. (2008) underwent reoperations because of DRUJ re-instability (*n* = 13), ECU tendonitis or superficial ulnar nerve pain respectively. No re-operations other than for DRUJ-re-instability were presented in the study by Luchetti et al. (2014).

Repair and re-insertion techniques were used in all patients included in the two studies. The immobilization time after surgery was somewhat different in the two studies; 5 ± 2 weeks in the study by Anderson et al., (2008) and 5 (long splint) + 4 (wrist splint) weeks in the study by Luchetti et al. (2014). Different time from injury to surgery was present – within 4 months of date of injury in the study by Anderson et al. (2008), and 11 (open surgery) - 13 (arthroscopic surgery) months in the study by Luchetti et al. (2014).

#### Quality assessment

There was substantial underreporting of important items in both studies. The methodological quality of the included articles showed high risk of bias in most items, except for blinding of outcome assessment, incomplete data outcome and selective reporting - where the risk of bias was unknown or low (Table 3).

**Table 3:** Results of the risk of bias assessment

Intervention	Author and Year	Random sequence generation	Allocation concealment	Blinding of participants and personnel	Blinding of outcome assesssment	Incomplete data outcome	Selective reporting
Open vs Arthroscopic	Anderson et al. 2008	High	High	High	Unclear	Low	Low
	Luchetti et al. 2014	High	High	High	Unclear	High	High

The included articles displayed some heterogeneity regarding participants, diagnostic methods, and study design. The attrition bias (lost to follow up from primary cohort) was high (42%) in the study by Luchetti et al. (2014), and low (1%) in the study by Anderson et al. (2008). Preoperative instability of the DRUJ was noted in 100% of the patients in the study by Luchetti et al., and in 36% in the study by Anderson et al. Inclusion and exclusion criteria differed slightly between the two studies and there were heterogenic cohorts in the included studies, making comparisons difficult. There were mixed cohorts, in terms of injury types (classification; TFCC injury 1B, 1C and/or 1D not clear) in one of the included studies (Anderson et al. 2008).

In both included studies, more arthroscopic repairs were performed in the later years of the studies, rendering shorter follow up time in arthroscopically treated patients. The two included studies were of level of evidence (LoE) III, according to the Oxford Centre for Evidence-Based Medicine rating system.

### Discussion

This systematic review of repair of TFCC injuries found comparable outcomes between open and arthroscopic surgery in terms of DRUJ re-instability and functional scores. It is noteworthy that the broad and systematic literature search only identified two cohort studies which compared open and arthroscopic surgery. The results suggest that open surgery might lead to a higher rate of extensor carpi ulnaris (ECU) tendinitis and neuroma. These results are probably due to larger surgical incisions in open techniques, with the need for incisions into or close to tendon sheets and capsule. In general, the numbers of studies regarding TFCC surgery reporting adequate information of all different complication rates (ECU tendinitis, neuroma and pain) is low. Instead, technical reports dominate the literature. Many different surgical techniques in terms of repair of the TFCC have been presented in the literature the last decades (Cooney et al. 1994; Garcia-Elias et al. 2003; Atzei et al. 2008; Bednar and Osterman 1994; Carlsen et al. 2009; Tang et al. 2013; Adams 2000; Sagerman and Short 1996; Trumble et al. 1996). Comparative studies are rare, and a systematic review and assessment of the overall results has so far been lacking.

Interestingly, women seem to have a higher risk of complications. Some studies (Anderson et al. 2008) stress that there is a higher re-operation rate for females, which can be compared to knee ACL reconstruction (Andernord et al. 2015). Anderson et al. (2008) displayed a 5 times higher rate of re-operation in female patients. The re-operation rate in TFCC/DRUJ surgery although far exceeds the rate in ACL reconstruction, by 4–5 times.

One study in the literature presents that outcome after arthroscopic suture of TFCC to capsule only has shown that there are worse outcome in older patients (Ruch and Papadonikolakis 2005). This is not described in studies with re-insertion techniques. One study of arthroscopic re-insertion of the TFCC surprisingly showed that there is *not* a worse outcome when ulnar positive variance exists (Kim et al. 2013). Papapetropoulos et al. (2010) and Reiter et al. (2008) have shown the same in terms of TFCC suture to capsule. This is in contrast with the common perception and opinion.

Information about ulnar variance is lacking in the two studies of comparison between open and arthroscopic repair of the TFCC included in this systematic review (Anderson et al. 2008; Luchetti et al. 2014), as well as age dependent outcome. Five Wafer resections were, however, performed in the open repair group in the study by Luchetti et al. (2014).

Evidence is lacking to support aggressive early surgical management when TFCC tears, especially partial ones, are diagnosed in association with distal radius fractures in adults (Mrkonjic et al. 2012). Persistant dorso-ulnar wrist pain in relation to powerful rotatory hand movement or lifting heavy objects, however, often motivates surgical treatment. The two included studies in the present systematic review used conservative treatment (splint, NSAIDs, physiotherapy) for at least 3 months before TFCC surgery. Furthermore, different injury-to-surgery intervals were reported. The majority of patients received treatment within 4 months of injury in the study by Anderson et al. (2008) and within 11 months (open) – 13 months (arthroscopic) in the study by Luchetti et al. (2014). This could have influenced the diagnostics, the rate and grade of DRUJ laxity - and its appearance - and the selection/inclusion of patients. The preoperative laxity of the DRUJ was different in the two studies with 36% in the study by Anderson et al. (2008), compared to 100% of the patients in the study by Luchetti et al. (2014). Thus, the DRUJ re-instability was relatively high in the study by Anderson et al. (2008).

The two included studies were of level of evidence (LoE) III, according to the rating system by Oxford Centre for Evidence-Based Medicine.

The follow-up criteria in many of the publications about TFCC surgery are sometimes not focused on the clinically most important findings. A recommendation is to use DRUJ re-instability as the primary measure outcome in all studies dealing with patients with TFCC injuries and concomitant DRUJ instability treated by re-insertion and MMWS or PRWE (most dedicated to wrist function) as the most valuable secondary measure outcomes. Preoperative patient scores and data are essential to compare with the postoperative outcome in order to know the efficacy of the surgical technique and to evaluate if operation is superior to non-surgical treatment.

To be able to recommend surgery and what kind of procedure in specific cases, larger studies with a randomized prospective study design (RCTs), equivalent studies or comparative studies are needed. RCTs in this area are difficult to perform, in terms of the rather few patients operated on by TFCC repair and the calculated long time needed to perform such study. Different techniques and modifications of TFCC surgery are still frequently introduced and described. Up to this date, the patient’s needs and the surgeon’s specific skills seem to have had a great impact on choosing type of surgical treatment for TFCC with- and without concomitant DRUJ instability. The choice of surgical technique still seems to be based on the discretion and preferences of the individual surgeon and the choice of open or arthroscopic TFCC repair is surgeon dependent.

Reoperation- and complication rate in TFCC surgery could be regarded as rather high, especially compared with the results in the most common orthopaedic ligament reconstruction area – ACL reconstruction (Andernord et al. 2015).

Some questions could be asked. *How strong are in fact our treatment recommendations for patients with TFCC injuries and DRUJ instability*? Only four studies (Anderson et al. 2008; Luchetti et al. 2014; Chou and Lee 2001; Nakamura et al. 2011) of comparison between open and arthroscopic TFCC repair were identified in the search strategies among all publications between 1985 and 2016. Two of them (Chou and Lee 2001; Nakamura et al. 2011) were excluded due to insufficient data and were classified as LoE V. There is an immense lack of comparison studies in the area and the evidence of recommending either open or arthroscopic surgery is still lacking.

*Which outcome measures should be included in future studies?*

Performing studies of TFCC injury with DRUJ instability and not evaluating the instability and re-instability rate pre- and postoperatively is suboptimal. Performing studies without reporting rates of complications and satisfaction is non-informative for future operative guidelines.

There are several important limitations to this systematic review. Only an electronic search was performed. However, the three most important biomedical databases (PubMed, Embase, and the Cochrane Library) were used. Statistical analysis of the data for the purpose of a meta-analysis was not possible due to heterogeneity among the included studies and because of the few studies included. There were no studies with true blinding of the authors. The power of this study is affected due to included articles displaying high risk of bias and heterogeneity regarding participants, diagnostic methods, and study design.

Important strengths were the extensive literature search in three of the largest medical databases, the broad search strategies and the application of strict inclusion and exclusion criteria. Moreover, the PRISMA statement guided the extraction and reporting of data.

## Conclusions

This systematic review found comparable results between open and arthroscopic repair of the TFCC, in terms of DRUJ re-instability and functional outcome scores. There is insufficient evidence to recommend one technique over the other in clinical practice. There is an immense lack of comparison studies with high level of evidence in the area of wrist ligament repair and reconstruction, including TFCC-injuries and DRUJ-instability.

## Appendix

### Search strategies

Database: PubMed.

Date: 2016–05-26.

No of results: 612 ref.

Search updated: 2017–03-21: 41 ref.

**Table:**

Search	Query	Items found
#51	Search #41 NOT #46 Filters: Publication date from 1985/01/01; Swedish; Norwegian; English	612
#47	Search #41 NOT #46	768
#46	Search Editorial[ptyp] OR Letter[ptyp] OR Comment[ptyp]	1480954
#41	Search #36 NOT #40	777
#40	Search #37 OR #38 OR #39	4265624
#39	Search animal[ti] OR animals[ti] OR cadaver*[ti]	112070
#38	Search ((cadaver[mh]) NOT (cadaver[mh] AND humans[mh]))	4491
#37	Search ((animals[mh]) NOT (animals[mh] AND humans[mh]))	4215235
#36	Search #12 AND #35	794
#35	Search #17 OR #34	399096
#34	Search arthroscop*[tiab] OR open[tiab]	395330
#17	Search “Arthroscopy”[Mesh]	18415
#12	Search #8 AND #11	2118
#11	Search #9 OR #10	3881200
#10	Search “Surgical Procedures, Operative”[Mesh]	2640423
#9	Search reconstructive[tiab] OR reconstruction[tiab] OR repair[tiab] OR suture[tiab] OR sutures[tiab] OR reinsertion[tiab] OR surgery[tiab] OR surgical[tiab] OR surgery[sh]	2680099
#8	Search triangular fibrocartilage complex OR triangular fibrocartilage ligament OR triangular fibro-cartilage complex OR triangular fibro-cartilage ligament OR distal radioulnar joint OR (wrist[tiab] AND ligament[tiab]) OR (wrist[tiab] AND ligaments[tiab]) OR tfcc[tiab] OR druj[tiab]	3193

Database: Embase (OvidSP) 1974 to 2016 May 23.

Date: 2016–05-26.

No of results: 209 ref.

Search updated: 2017–03-21: 60 ref.

Database(s): Embase 1974 to 2017 March 20.

**Table:**

#	Searches	Results
1	(reconstructive or reconstruction or repair or suture or sutures or reinsertion or surgery or surgical).ti,ab.	2,173,970
2	exp ligament surgery/	5352
3	1 or 2	2,174,910
4	((triangular adj3 fibro?cartilage adj3 complex) or (triangular adj3 fibro?cartilage adj3 ligament)).ti,ab.	651
5	(distal adj3 radio?ulnar adj3 joint).ti,ab.	1048
6	(wrist adj5 ligament?).ti,ab.	377
7	4 or 5 or 6	1822
8	3 and 7	812
9	arthroscopy/ or exp. wrist arthroscopy/	15,586
10	(arthroscop$ or open).ti,ab.	489,507
10	(arthroscop$ or open).ti,ab.	489,507
11	9 or 10	493,437
12	8 and 11	304
13	(animal or animals or cadaver$).ti.	124,117
14	(animal not (animal and human)).sh.	1,325,044
15	13 or 14	1,417,596
16	12 not 15	298
17	limit 16 to (embase and (danish or english or norwegian or swedish) and yr. = “1985 -Current” and (article or conference paper or note or “review”))	209

Database: The Cochrane Library.

Date: 2016–05-26.

No of results: 47 ref.

Search updated: 2017–03-21: 4 ref. (only in Trials).

*Cochrane review: 21.*

*Other reviews: 1.*

*Trials: 25 + 4 from updated search.*

*Technology assessments: -.*

*Economic evaluations: -.*

**Table:**

ID	Search	Hits
#1	reconstructive or reconstruction or repair or suture or sutures or reinsertion or surgery or surgical:ti,ab,kw (Word variations have been searched)	119,374
#2	MeSH descriptor: [Surgical Procedures, Operative] explode all trees	110,760
#3	#1 or #2	176,755
#4	triangular fibrocartilage complex or triangular fibrocartilage ligament or triangular fibro-cartilage complex or triangular fibro-cartilage ligament or distal radioulnar joint or (wrist and ligament?)	69
**#5**	**#3 and #4**	**47**

Database: PubMed.

Date: 2017–12-27.

No of results: 48 ref.

**Table:**

Search	Query	Items found
#26	Search (#19 NOT #20) Filters: Publication date from 2017/03/21 to 2017/12/31; English; Norwegian; Swedish	48
#21	Search (#19 NOT #20)	958
#20	Search (editorial[ptyp] OR letter[ptyp] OR comment[ptyp])	1,594,371
#19	Search (#14 NOT #18)	969
#18	Search (#15 OR #16 OR #17)	4,462,809
#17	Search (animal[ti] OR animals[ti] OR cadaver*[ti])	118,447
#16	Search ((cadaver[mh] NOT (cadaver[mh] AND humans[mh]))	4791
#15	Search ((animals[mh] NOT (animals[mh] AND humans[mh]))	4,408,539
#14	Search (#10 AND #13)	992
#13	Search (#11 OR #12)	445,171
#12	Search (arthroscop*[tiab] OR open[tiab])	441,135
#11	Search arthroscopy[MeSH Terms]	20,301
#10	Search (#6 AND #9)	3512
#9	Search (#6 OR #7)	2,892,064
#8	Search surgical procedures, operative[MeSH Terms]	2,815,328
#7	Search (reconstructive[tiab] OR reconstruction[tiab] OR repair[tiab] OR suture[tiab] OR sutures[tiab] OR reinsertion[tiab] OR surgery[tiab] OR surgical[tiab] OR surgery[sh])	2,890,746
#6	Search (triangular fibrocartilage complex OR triangular fibrocartilage ligament OR triangular fibro-cartilage complex OR triangular fibro-cartilage ligament OR distal radioulnar joint OR (wrist[tiab] AND ligament[tiab]) OR (wrist[tiab] AND ligaments[tiab]) OR tfcc[tiab] OR druj[tiab])	3512

Database: Embase (OvidSP).

Date: 2017–12-27.

No of results: 17 ref.

**Table:**

#	Searches	Results
1	(reconstructive or reconstructionor repair or suture or sutures or reinsertion or surgery or surgical).ab,ti.	2,085,704
2	exp ligament surgery/	13,686
3	1 or 2	2,092,271
4	((triangular adj3 fibro?cartilage adj3 complex) or (triangular adj3 fibro?cartilage adj3 ligament)).ti,ab.	776
5	(distal adj3 radio?ulnar adj3 joint).ti,ab.	1184
6	(wrist adj5 ligament?).ti,ab.	422
7	4 or 5 or 6	2067
8	3 and 7	801
9	arthroscopy/ or wrist arthroscopy/	17,438
10	(arthroscop$ or open).ti,ab.	564,967
11	9 or 10	569,262
12	8 and 11	300
13	(animal or animals or cadaver$).ti.	129,264
14	(animal not (animal and human)).sh.	1,387,774
15	13 or 14	1,483,818
16	12 not 15	294
17	from 16 keep 2–7,13–15,18–19,28,36–37,41–43 limit to english, swedish, norwegian) and yr. = 2017	17

Database: The Cochrane Library.

Date: 2017–12-27.

No of results: 0 ref.

**Table:**

ID	Search	Hits
#1	Reconstructive or reconstruction or repair or suture or sutures or reinsertion or surgery or surgical:ti,ab,kw: publication year 2017	12,108
#2	MeSh descriptor: [Surgical Procedures,Operative] explode all trees: publication year 2017	121,196
#3	#1 or #2: publication year 2017	12,731
#4	Triangular fibrocartilage complex or triangular fibrocartilage ligament or triangular fibro-cartilage complex or triangular fibro-cartilage ligament or distal radioulnar joint or (wrist and ligament?): Publication year 2017	0
#5	#3 and #4	0
